# Cutaneous Manifestations of Systemic Lupus Erythematosus and Their Correlation With Cardiac Involvement

**DOI:** 10.7759/cureus.76478

**Published:** 2024-12-27

**Authors:** Romasa Zeb, Daniela Valentina Combariza Chinome, Maria Chacon, Taranpreet Singh, Machineni Meghana Chowdary, Claudia Jeanette Manzanares Vidals, Sunjida Mehnaz, Andres Felipe Torres Medina, Prashanthi Sarayu Gadde, Raaj Pawan Kumar Lingamgunta, Aly Barakat, Manju Rai

**Affiliations:** 1 Internal Medicine, Army Medical College, Rawalpindi, PAK; 2 Internal Medicine, New Granada Military University, Bogota, COL; 3 Internal Medicine, Universidad de Especialidades Espíritu Santo, Guayaquil, ECU; 4 Internal Medicine, Mahatma Gandhi Mission (MGM) Medical College and Hospital, Navi Mumbai, IND; 5 Internal Medicine, Siddhartha Medical College, Vijayawada, IND; 6 Internal Medicine, General Hospital of Toluca Dr. Nicolas San Juan, Toluca, MEX; 7 Internal Medicine, Medway Maritime Hospital, Kent, GBR; 8 Internal Medicine, Universidad de Ciencias Aplicadas y Ambientales U.D.C.A, Bogota, COL; 9 Internal Medicine, MNR Medical College, Hyderabad, IND; 10 Internal Medicine, NRI Medical College & General Hospital, Guntur, IND; 11 Internal Medicine, Medway NHS Foundation Trust, Kent, GBR; 12 Biotechnology, Shri Venkateshwara University, Gajraula, IND

**Keywords:** autoimmune disease and heart complications, biologics in lupus management, cardiac involvement in sle, cutaneous manifestations, immunosuppressive therapy in sle, lupus skin lesions, multidisciplinary care in sle, skin-cardiac correlation in lupus, systemic lupus erythematosus (sle)

## Abstract

Systemic lupus erythematosus (SLE) is a chronic autoimmune disorder characterized by widespread immune dysregulation that affects multiple organ systems, including the skin and cardiovascular system. The crosstalk between different cell death pathways-such as apoptosis, necroptosis, and neutrophil extracellular trap (NETosis), plays a pivotal role in the pathogenesis of SLE, influencing both cutaneous and cardiac manifestations. Cutaneous lupus erythematosus (CLE) is one of the most common early signs of SLE, affecting up to 80% of patients. CLE presents in several forms, including acute, subacute, and chronic lesions, each with varying degrees of association with systemic disease. Cardiac involvement, although often underrecognized, significantly contributes to morbidity and mortality in SLE patients, manifesting as pericarditis, myocarditis, valvular disease, and accelerated atherosclerosis. Emerging research suggests that these cutaneous and cardiac manifestations may be connected through shared immune mechanisms, including immune complex deposition, endothelial dysfunction, and chronic inflammation driven by cytokines such as Interleukin-6 (IL-6) and tumor necrosis factor-alpha (TNF-α). The severity of skin involvement may correlate with an increased risk of cardiovascular events, underscoring the importance of early diagnosis and a multidisciplinary approach to treatment. This review explores the crosstalk among cell death pathways in SLE and examines how these pathways contribute to both cutaneous and cardiac manifestations. Furthermore, it highlights the clinical implications of this crosstalk and discusses potential therapeutic strategies aimed at modulating these cell death pathways to improve patient outcomes. Challenges and gaps in current research are also addressed, emphasizing the need for further investigation into these complex interactions.

## Introduction and background

Systemic lupus erythematosus (SLE) is a chronic, multisystem autoimmune disorder characterized by the production of autoantibodies against nuclear antigens, leading to immune complex deposition and persistent inflammation in various organs. SLE has an incidence rate of 17 to 48 cases per 100,000 individuals, predominantly affecting women, with a female-to-male ratio of 9:1, and it often involves the skin, joints, kidneys, and cardiovascular system [1]. Despite advances in diagnosis and therapeutic options, SLE remains a significant burden, with a high degree of morbidity and mortality [2]. Early diagnosis and effective management are essential, especially as the disease may initially present with relatively mild symptoms before progressing to involve critical organs [3].

Cutaneous involvement is among the most frequent early presentations of SLE, occurring in up to 80% of patients [4]. Cutaneous lupus erythematosus (CLE) is broadly classified into lupus-specific and lupus-nonspecific lesions [5]. Lupus-specific cutaneous manifestations include acute, subacute, and chronic cutaneous lupus erythematosus (ACLE, SCLE, and CCLE, respectively), each with distinct clinical and histopathological features. Commonly presenting as the classic butterfly (malar) rash, ACLE is often associated with more severe systemic disease [5]. SCLE (Annular and Papulosquamous variants) and CCLE, including discoid lupus erythematosus (DLE), have a prolonged course and are also important in assessing systemic disease risk.

Nonspecific skin lesions in SLE include photosensitivity, oral ulcers, alopecia (typically non-scaring diffuse alopecia), and Raynaud phenomenon [6]. These nonspecific features are often present during the active phases of the disease and are part of the diagnostic criteria established by the American College of Rheumatology (ACR). Identifying and understanding these cutaneous markers can facilitate early diagnosis and provide insights into the progression of systemic involvement.

Cardiac involvement in SLE, though sometimes under-recognized, is a significant contributor to increased morbidity and mortality. Almost all cardiac structures can be affected in SLE, including the pericardium, myocardium, endocardium, valves, and coronary arteries [7]. Pericarditis is the most common cardiac manifestation, with presentations ranging from asymptomatic to symptomatic pericardial effusion [8]. Valvular involvement, especially Libman-Sacks endocarditis, is another key feature of cardiac lupus [9]. The presence of antiphospholipid antibodies (aPL) in SLE patients is closely linked to valvular disease and increased risk of thrombotic events, such as myocardial infarction and stroke [9-10]. The correlation between cutaneous manifestations and cardiac complications may be due to shared inflammatory pathways, such as the involvement of T helper 1 and T helper 17 cells (Th1/Th17) responses and the release of pro-inflammatory mediators that contribute to both cutaneous and cardiac pathology [11].

The objective of this narrative review is to explore the correlation between cutaneous manifestations of SLE and associated cardiac involvement. By examining shared pathophysiological pathways and clinical evidence, this review aims to highlight the importance of early identification and multidisciplinary management of these manifestations to improve patient outcomes.

## Review

Pathophysiology of SLE

**Figure 1:**
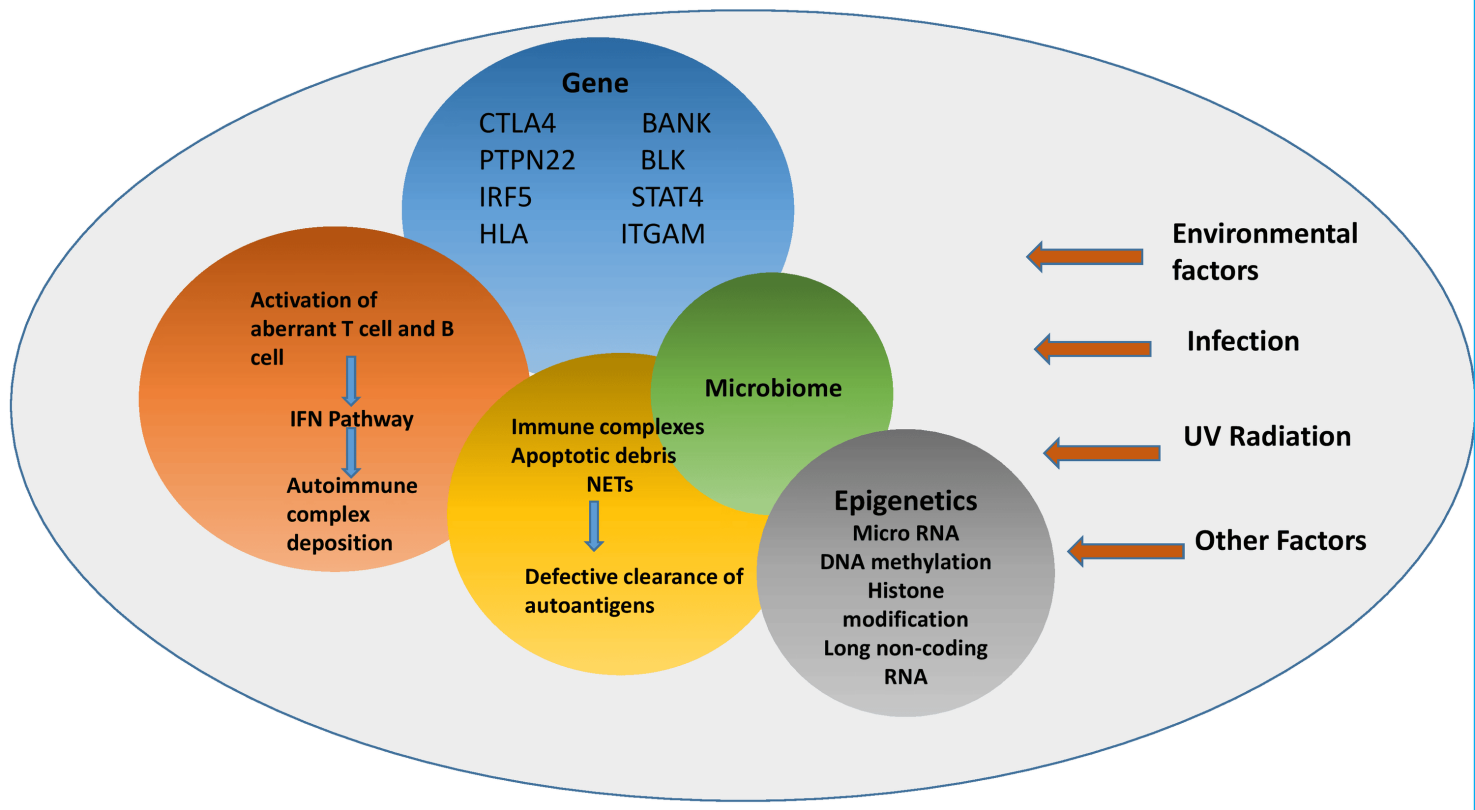
Mechanism of SLE pathogenesis The pathogenesis of SLE involves a complex interaction of factors, including genetic predisposition, epigenetic dysregulation, defective clearance of autoantigens, deposition of autoimmune complex, and dysbiosis of microbiota. Environmental risks such as ultraviolet light (UV) and infections accelerate the production of apoptotic debris and subsequently activate the autoinflammatory cascade in multiple ways. In addition, other mechanisms such as hormonal milieu and X chromosome abnormalities have been reported to contribute to the female susceptibility to SLE. CTLA4: Cytotoxic T-lymphocyte–associated antigen 4; PTPN22: Tyrosine-protein phosphatase non-receptor type 22; IRF5: Interferon Regulatory Factor 5; HLA: Human leukocyte antigens; BLK: B-lymphoid tyrosine kinase; STAT4: Signal transducer and activator of transcription 4; ITGAM: Integrin subunit alpha M; IFN: Interferon; NET: Neutrophil extracellular traps; RNA: Ribonucleic acid; SLE: Systemic lupus erythematosus; BANK: B-cell scaffold protein with ankyrin repeats Image credits: Aly Barakat

The pathogenesis of SLE involves a complex crosstalk of genetic, immunological, and environmental factors, contributing to immune dysregulation (Figure 1).

Genetic and Epigenetic Factors

Genetic susceptibility plays a key role in the development of SLE. Several genes within the human leukocyte antigen (HLA) region, particularly HLA-DR2 and HLA-DR3, have been strongly associated with an increased risk of developing SLE. Additionally, non-HLA genes such as PTPN22, STAT4, and IRF5 have been implicated in disease susceptibility. These genes contribute to immune system dysfunction, enhancing the production of autoantibodies and pro-inflammatory cytokines [12].

In addition to genetic predisposition, epigenetic modifications also contribute to SLE pathogenesis. Changes in DNA methylation, histone modifications, and non-coding RNAs, especially microRNAs, have been shown to regulate immune responses, leading to aberrant expression of pro-inflammatory genes [13].

Defective Clearance of Apoptotic Cells

A critical abnormality in SLE is the defective clearance of apoptotic cells. Under normal circumstances, apoptotic cells are swiftly removed to prevent immune activation. However, there is impaired clearance in SLE, leading to the accumulation of apoptotic debris, including nuclear material. This debris acts as a source of autoantigens, driving the production of autoantibodies, such as anti-dsDNA, and triggering an autoimmune response [14]. The chronic presence of these immune complexes is central to disease activity and tissue damage.

Neutrophil Extracellular Traps (NETs)

Recent studies have highlighted the role of neutrophil extracellular traps (NETs) in SLE. NETs are composed of decondensed chromatin fibers mixed with antimicrobial proteins that are released by neutrophils during infections. In SLE, neutrophils undergo excessive NETosis, leading to the release of nuclear material that serves as a potent autoantigen. This contributes to immune activation and tissue inflammation, exacerbating disease progression [15].

Complement System Dysfunction

The complement system is an integral part of immune complex clearance. In SLE, defects in the complement system, particularly deficiencies in C1q, C2, and C4, compromise the removal of immune complexes. This results in their deposition in various tissues, such as the kidneys, skin, and joints, resulting in local inflammation and damage. Inadequate complement function further exacerbates the formation of immune complexes, perpetuating the cycle of inflammation [16].

Toll-Like Receptor (TLR) Activation

Toll-like receptors (TLRs), particularly TLR-7 and TLR-9, recognize nucleic acids (RNA and DNA, respectively) and are hyperactive in patients with SLE. These receptors, especially in plasmacytoid dendritic cells, promote the production of type I interferons. This interferon signature is a central feature of SLE and is responsible for driving B-cell activation and autoantibody production, exacerbating immune dysregulation [17].

Environmental Triggers

Environmental factors play a significant role in triggering SLE in genetically predisposed individuals. Ultraviolet (UV) light is one of the most well-recognized triggers, as it promotes the release of nuclear antigens from damaged keratinocytes, which can then act as targets for autoantibodies. Additionally, infections, particularly with Epstein-Barr Virus (EBV), and environmental exposures such as smoking and certain drugs (e.g., procainamide, hydralazine) can initiate or exacerbate SLE flares. These environmental triggers contribute to immune dysregulation by activating autoreactive lymphocytes [18].

B-Cell Hyperactivity

One of the central features of SLE is B-cell hyperactivity. In SLE, B-cells lose tolerance and produce large amounts of autoantibodies, such as anti-dsDNA and anti-Sm antibodies. This B-cell overactivity is driven by abnormal T-cell help, cytokines (such as BAFF), and type I interferons. Autoantibodies form immune complexes with self-antigens, contributing to tissue inflammation and damage [19].

Pathophysiological Mechanisms Linking SLE and Cardiac Involvement

**Figure 2:**
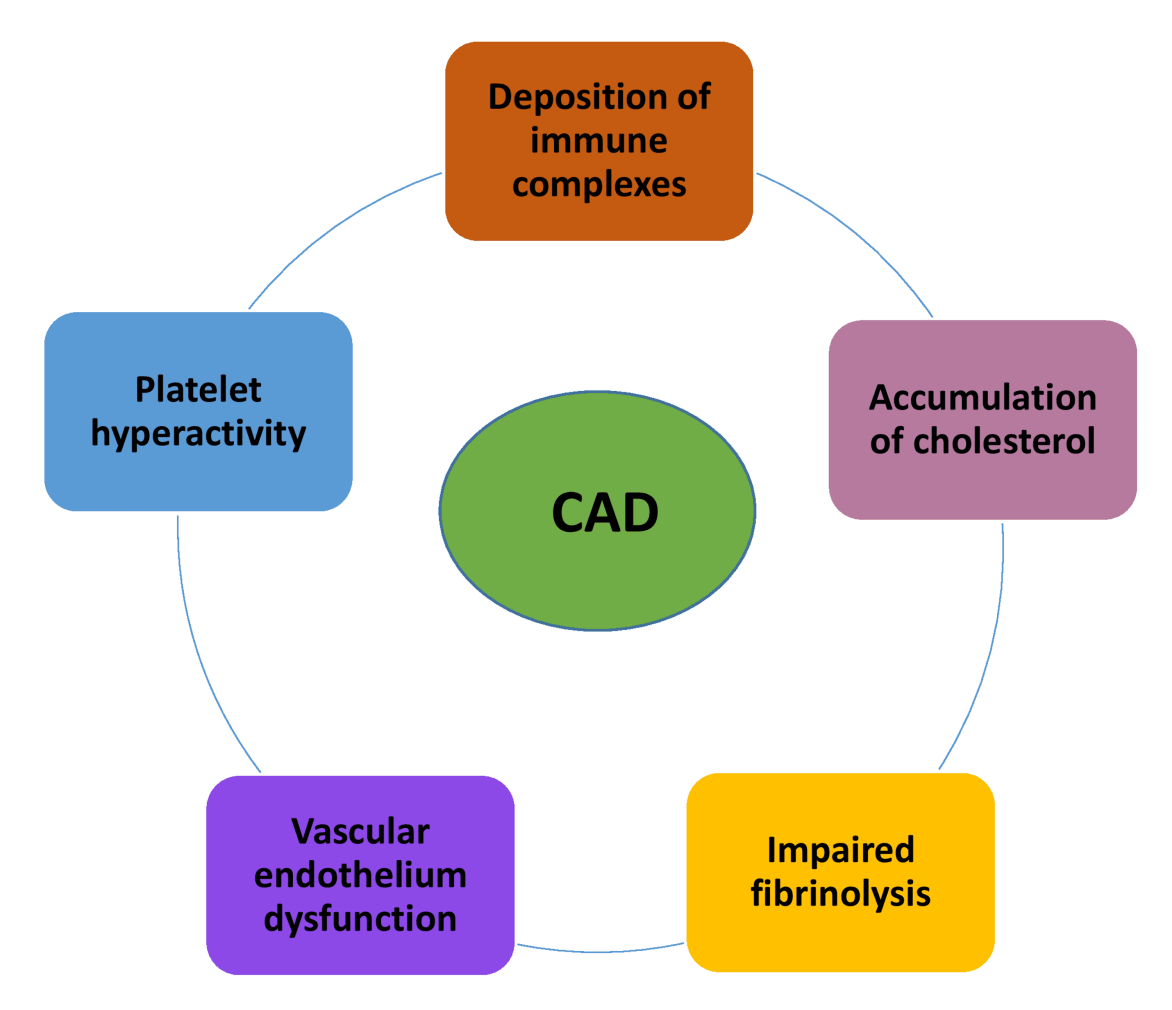
CAD pathophysiology in SLE. The figure depicts the pathophysiological mechanisms involved in developing CAD in SLE. It demonstrates how the deposition of immune complexes triggers inflammatory processes within the vascular walls, contributing to endothelial damage. Additionally, cholesterol accumulation promotes atherosclerotic plaque formation, while impaired fibrinolysis hinders the breakdown of clots, increasing the risk of thrombosis. Dysfunction of the vascular endothelium further exacerbates vascular injury, and platelet hyperactivity enhances clot formation, leading to potential vascular occlusion. Together, these mechanisms contribute to the heightened risk of CAD in SLE patients. CAD: Coronary artery disease; SLE: Systemic lupus erythematosus Image credits: Raaj Pawan Kumar Lingamgunta

SLE is characterized by immune system dysregulation that promotes systemic inflammation, leading to tissue damage across multiple organs, including the heart [20]. A key mechanism is endothelial cell dysfunction (Figure 2), which triggers the expression of the lectin-like oxidized low-density lipoprotein receptor 1 (LOX-1) [21]. This receptor, in turn, initiates endothelial damage and activates pro-inflammatory cytokines such as tumor necrosis factor-alpha (TNF-α), interleukin-6 (IL-6), and interleukin-12 (IL-12), which attract monocytes and promote vascular injury [22]. Additionally, the overactivation of CD4+ T lymphocytes exacerbates vascular damage through interferon-1 signaling, contributing to the formation of thrombi [22].

**Figure 3:**
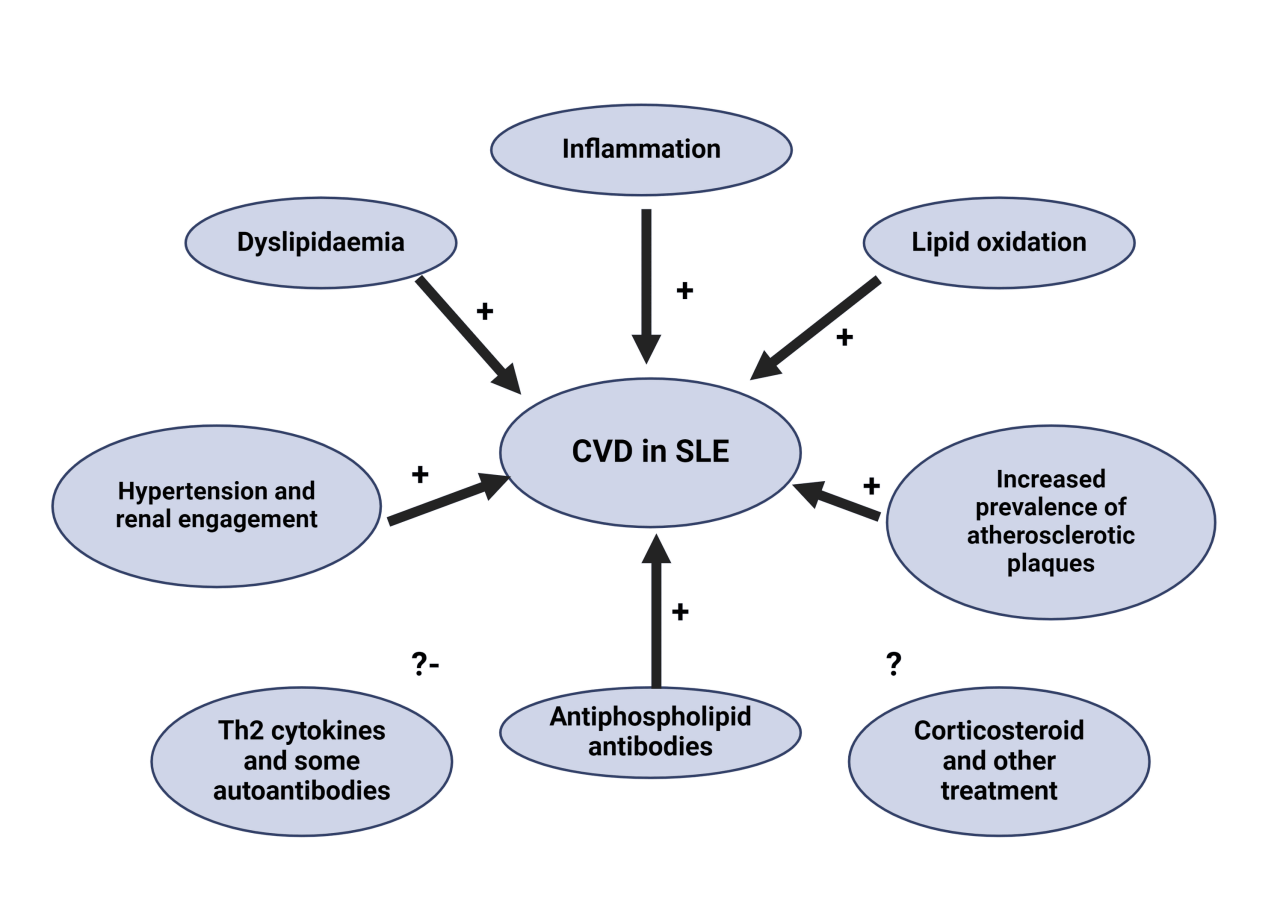
CVD in SLE etiology: Pathophysiological mechanisms contributing to CVD in SLE. This diagram represents the various mechanisms contributing to cardiovascular disease development in patients with SLE. Inflammation, dyslipidemia, lipid oxidation, hypertension with renal involvement, and antiphospholipid antibodies are all known to contribute to CVD progression in SLE. Additionally, the increased prevalence of atherosclerotic plaques further exacerbates cardiovascular risk. The roles of Th2 cytokines, autoantibodies, corticosteroids, and other treatment modalities remain controversial. Together, these factors contribute to the elevated cardiovascular risk observed in SLE patients. CVD: Cardiovascular diseases; SLE: Systemic lupus erythematosus; Th2: T helper 2 Image Credits: Prashanthi Sarayu Gadde

Several forms of programmed cell death, including necroptosis, pyroptosis, NETosis, and ferroptosis, are upregulated in SLE patients with cardiac involvement [23]. These processes amplify the immune response, further driving inflammation and promoting damage to cardiac tissues. Autoantibodies, such as anticardiolipin, anti-beta-2 glycoprotein, and lupus anticoagulant, also contribute to a procoagulant state, exacerbating the risk of thrombus formation and valvular damage (Figure 3) [24]. Specifically, these antibodies can injure endothelial cells, particularly on the mitral valve, leading to non-bacterial thrombotic endocarditis (NBTE).

**Figure 4:**
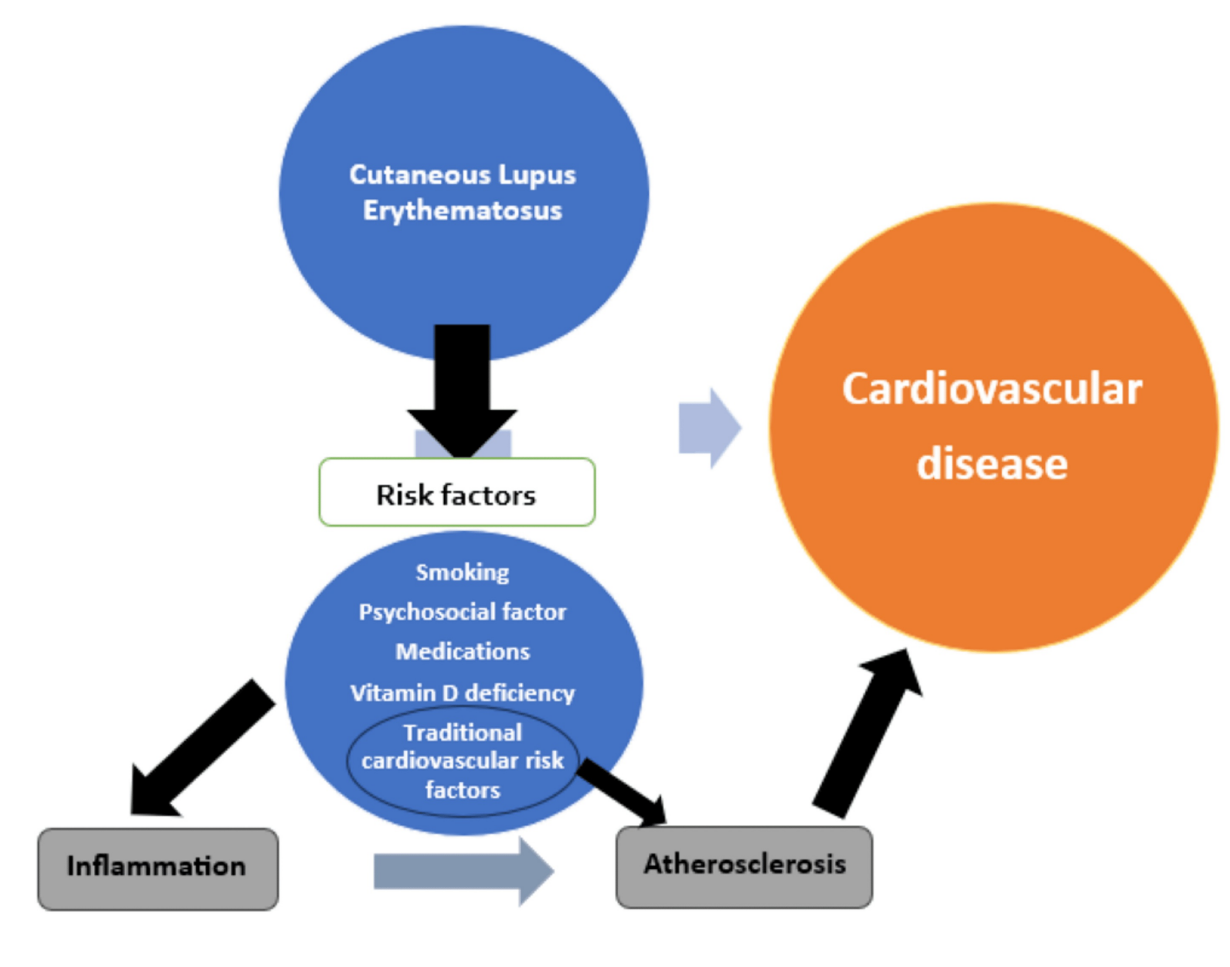
CLE-related pathways to CVD This diagram illustrates the potential mechanisms linking CLE to CVD. CLE contributes to an increased cardiovascular risk in combination with other multiple factors, including smoking, psychosocial stress, medications, vitamin D deficiency, and traditional cardiovascular risk factors. These risk factors lead to inflammation and atherosclerosis, both of which play critical roles in the development of cardiovascular disease in patients with CLE. The interconnection between inflammatory pathways and the progression of atherosclerosis highlights the complex interaction between CLE and CVD. CLE: Cutaneous lupus erythematosus; CVD: Cardiovascular disease Image Credits: Sunjida Mehnaz

Additionally, cardiovascular disease (CVD) in SLE is driven by traditional risk factors like hypertension, smoking, and hypercholesterolemia, often exacerbated by immunosuppressive therapy (Figure 4).

Hypotheses on Shared Pathophysiological Pathways

The extent and severity of skin involvement may serve as a predictor for cardiac manifestations. Recent studies have reinforced the association between cutaneous manifestations and cardiac involvement in SLE. A large cohort study by Olbrich et al. (2021) found that patients with discoid lupus erythematosus (DLE) had a significantly higher prevalence of pericarditis and myocarditis compared to patients without these skin manifestations [25]. Similarly, a 2021 study by Gamal et al. identified a higher incidence of cardiovascular disease, including pericarditis and myocarditis, in SLE patients presenting with skin vasculitis or livedo reticularis [26]. These findings suggest that specific skin lesions may be predictive of a heightened risk for cardiac complications in SLE, reinforcing the need for cardiovascular monitoring in patients with significant skin involvement.

The correlation between skin and heart manifestations in SLE is thought to be driven by shared immune-mediated mechanisms. Both cutaneous and cardiac manifestations may result from immune complex deposition and chronic inflammation, which are central to the pathogenesis of SLE. Immune complexes, composed of self-antigens and autoantibodies, deposit in the skin and cardiac tissues, leading to complement activation, recruitment of inflammatory cells, and tissue damage. It has been demonstrated that circulating immune complexes in SLE patients contribute to endothelial dysfunction, a common denominator in both cutaneous vasculitis and cardiac disease [3].

Furthermore, pro-inflammatory cytokines such as interleukin-6 (IL-6) and tumor necrosis factor-alpha (TNF-α) are upregulated in SLE, contributing to the systemic inflammation that underpins both cutaneous and cardiac lesions. Chronic inflammation has also been shown to accelerate atherosclerosis in SLE, which explains the increased cardiovascular risk in patients with severe skin involvement.

Severe cutaneous manifestations, including vasculitic lesions, bullous lupus, or widespread SCLE, are associated with a risk of myocarditis, pericarditis, and valvular disease. Recent studies by Uccello et al. (2024) have shown that patients with extensive skin flares are more likely to develop subclinical cardiac abnormalities, including left ventricular dysfunction and arrhythmias [27]. As such, dermatologic assessments in SLE are critical for identifying patients who may benefit from early cardiovascular screening and preventive interventions [27].

Cutaneous manifestations of SLE

While SLE often includes skin involvement, CLE can occur independently of systemic disease, manifesting solely in the skin. Approximately 70-85% of SLE patients experience cutaneous symptoms during the course of the illness, with CLE being the first presenting feature in about 25% of these patients [28].

ACLE manifests through both localized and generalized lesions. The most recognized localized lesion is the malar rash, often referred to as the "butterfly rash," which is characterized by symmetrical erythema and swelling over the cheeks and the bridge of the nose, typically sparing the nasolabial folds [28-30]. Malar rash is strongly associated with systemic involvement and may present at the time of diagnosis in 40-52% of SLE patients [31]. Generalized lesions of ACLE include maculopapular rashes, which predominantly affect sun-exposed areas of the body. Bullous lupus erythematosus (BLE), a subepidermal blistering condition, is another key feature of ACLE, affecting roughly 5% of SLE patients [30].

Histopathological findings in ACLE include vacuolar interface dermatitis, mild lymphocytic infiltrate, and dermal edema, reflecting active inflammation. These findings help distinguish ACLE lesions from similar conditions like sunburn, rosacea, or seborrheic dermatitis [30].

SCLE typically presents as a photosensitive rash with two predominant forms: annular (ring-shaped erythematous plaques) and papulosquamous (psoriasis-like scaly lesions). The rash commonly affects the upper trunk, neck, and shoulders, often sparing the face and scalp [30]. SCLE is known for its strong association with anti-Ro/SSA antibodies, which are present in many patients [30].

Histopathologically, SCLE shares features with other CLE forms, including interface dermatitis and epidermal atrophy. However, SCLE tends to show more pronounced epidermal changes compared to ACLE or DLE. Around 50% of SCLE patients may eventually meet the criteria for SLE, though these patients generally experience milder systemic disease [31].

CCLE encompasses several subtypes, the most common being Discoid Lupus Erythematosus (DLE). DLE presents as well-demarcated, erythematous, scaly plaques that typically heal with scarring and pigmentary changes. DLE lesions can be localized (confined to the face, scalp, and ears) or disseminated (involving the upper and lower trunk) [32]. The localized form is less likely to progress to SLE, whereas the disseminated form carries a higher risk [32].

Histopathological examination of DLE lesions reveals hyperkeratosis, follicular plugging, thickening of the basement membrane, and a dense lymphocytic infiltrate in the dermis [33]. These features reflect the chronic, scarring nature of DLE [33].

The potential progression from CLE to SLE underscores the importance of continuous monitoring. ACLE, particularly the malar rash, is most closely linked to systemic lupus, with frequent positivity for antinuclear antibodies (ANA) and anti-double-stranded DNA (ds-DNA) [4]. In contrast, DLE and other forms of CCLE tend to have a lower risk of systemic involvement, though patients with disseminated lesions or periungual involvement may face a higher risk [1].

The chronicity, scarring potential, and severity of CLE subtypes also guide therapeutic decisions. ACLE, especially when systemic involvement is present, may require systemic immunosuppressants such as corticosteroids or antimalarials [32]. In contrast, DLE may respond well to topical therapies, including corticosteroids or calcineurin inhibitors [34]. Early aggressive intervention in DLE is crucial to prevent scarring and long-term damage [34].

Histopathological analysis is integral in differentiating CLE subtypes from other dermatologic conditions, such as psoriasis, rosacea, and dermatomyositis, ensuring accurate diagnosis and treatment. For instance, vacuolar interface dermatitis in ACLE reflects active inflammation, while basal vacuolization and superficial lymphocytic infiltrates in malar rash help distinguish it from other causes of facial erythema [35]. In DLE, the presence of a dense lymphocytic infiltrate correlates with the scarring and chronicity of the disease [36].

Less common forms of CLE include lupus erythematosus profundus (LEP), chilblain lupus, and lupus tumidus (LET), each presenting unique clinical challenges. For instance, chilblain lupus is associated with cold exposure and presents as painful erythematous lesions on the digits [37]. While these forms are less likely to progress to systemic lupus, they require targeted management to prevent chronicity and tissue damage.

Clinical spectrum of cardiac manifestations in SLE

Pericarditis

Pericarditis is the most common cardiac manifestation in SLE, affecting 20-50% of patients [21]. Clinical presentation mirrors that of typical acute pericarditis, with pleuritic chest pain, dyspnea, fever, and a pericardial rub. Electrocardiogram (ECG) findings often include ST-segment elevation and pointed T waves. Pericardial effusions are usually mild, rarely leading to serious complications such as cardiac tamponade. Treatment typically includes corticosteroids, such as intramuscular triamcinolone or oral methylprednisolone for mild cases, with intravenous methylprednisolone reserved for severe cases [38]. Autopsy studies suggest that the prevalence of pericarditis may be even higher than reported clinically [39].

Myocarditis

Although myocarditis is now less common in SLE due to the early use of immunosuppressive therapies, it remains a serious manifestation [10,39]. Clinical symptoms include heart failure, tachycardia, and unexplained ECG changes. Prevalence is estimated between 7-10%, with those suffering from myositis being at a higher risk [10]. Patients often require aggressive treatment with immunosuppressants, including high-dose corticosteroids [10].

Valvular Disease and Libman-Sacks Endocarditis

Valvular abnormalities, particularly involving the mitral and aortic valves, are frequent in SLE (30-60%). Libman-Sacks endocarditis, a hallmark feature, involves the formation of sterile vegetations on valve surfaces [40]. Although often asymptomatic, these vegetations result in valvular dysfunction, causing dyspnea and left-sided heart failure, and they increase the risk of developing infective endocarditis [24,41]. 

Arrhythmias and Electrophysiological Abnormalities

Arrhythmias are common in SLE patients, particularly sinus tachycardia and atrial fibrillation. Prolonged QT intervals (10-40%) are also frequently observed, often correlating with high disease activity and longer disease duration [20,39].

CAD and Atherosclerosis

Coronary artery disease is a leading cause of mortality in patients with late-onset or long-standing SLE. These patients experience accelerated atherosclerosis, comparable to that seen in diabetes mellitus [39]. Chronic endothelial dysfunction, combined with prolonged exposure to inflammatory mediators and autoantibodies, accelerates the development of coronary artery disease [41]. The chronic use of corticosteroids, which are commonly prescribed for SLE, further exacerbates the risk by promoting hyperlipidemia, hyperglycemia, and hypertension [42]. In the early stages of the disease, coronary events may be linked to coronary arteritis, myocarditis, or thrombosis, while long-term SLE is associated with progressive coronary atherosclerosis [43].

Thrombotic Events

Thromboembolic complications are common in SLE, particularly in the presence of antiphospholipid antibodies [44]. These antibodies increase the risk of arterial and venous thrombosis, which can affect coronary arteries and lead to myocardial infarction.

Table 1 represents various cardiac complications, their clinical manifestations, and pathophysiology.

**Table 1:** Clinical manifestations and pathophysiology of various cardiac complications associated with SLE. SLE: Systemic lupus erythematosus

Clinical manifestation	Key characteristics	Associated pathophysiology
Pericarditis	Pleuritic chest pain, pericardial rub, dyspnea, fever, tachycardia, ECG changes (ST-segment elevation, peaked T waves), pericardial effusion	Inflammation of the pericardium, possibly mild effusions, immune complex deposition, and autoreactive T cells
Endocarditis	Non-bacterial thrombotic endocarditis, valvular vegetations (Libman-Sacks), potential dyspnea, and left-sided heart failure	Sterile vegetation on mitral and aortic valves, increased risk of infective endocarditis, valve thickening/dysfunction
Myocarditis	Cardiomegaly, heart failure, tachycardia, and unexplained EKG changes	Inflammatory infiltration of myocardium, autoantibodies targeting myocardial cells, immune complex deposition.
Valvular heart disease	Thickened mitral and aortic valves, potential dysfunction, and left-sided heart failure	Immune-mediated damage, valve thickening, and Libman-Sacks endocarditis lead to valve dysfunction.
Coronary disease	Thrombi, atherosclerosis, vasculitis causing obstruction of coronary arteries	Immune-mediated vasculitis accelerated atherosclerosis due to prolonged inflammation and chronic steroid use.
Other coronary manifestations	Coronary arteritis, myocarditis, coronary thrombosis, or embolization with spontaneous recanalization	Inflammatory damage to coronary vessels, thrombosis triggered by antiphospholipid antibodies
Arrhythmias and electrophysiological abnormalities	Sinus tachycardia, atrial fibrillation, prolonged QT interval	High disease activity, autoimmune attack on conductive tissue, chronic inflammation
Thrombotic Events	Venous/arterial thrombosis, myocardial infarction	Antiphospholipid antibodies causing hypercoagulability, endothelial dysfunction

Therapeutic approaches

Given the potential correlation between skin involvement and cardiac risk, therapeutic approaches should address both types of manifestations, with the goal of reducing systemic inflammation and preventing organ damage. Current treatments for cutaneous manifestations not only aim to control skin disease but also have implications for cardiac outcomes, particularly when aggressive systemic inflammation is involved.

Current Treatments for Cutaneous Manifestations and Their Effects on Cardiac Outcomes

Although there are only three FDA-approved medications for SCLE-corticosteroids, hydroxychloroquine, and belimumab, there are currently no specific FDA-approved medications for CLE. Pharmacological therapy, which encompasses topical and systemic treatments, is the accepted standard treatment for CLE. Preventive measures, such as the cessation of smoking, the elimination of photosensitizing medications, vitamin D supplementation, and sun protection, are also crucial adjuncts to disease management (Table 2) [45].

**Table 2:** The dermatological and cardiovascular effects of treatments commonly used for CLE and SLE underscore the interconnected nature of the disease's systemic impact. CLE: Cutaneous lupus erythematosus; SLE: Systemic lupus erythematosus; SCLE: Subacute cutaneous lupus erythematosus

Therapeutic Approach	Mechanism of Action	Effect on Cutaneous Manifestations	Effect on Cardiac Outcomes
Hydroxychloroquine (HCQ)	Anti-inflammatory, antithrombotic, reduces autoantibody production	The primary treatment for CLE improves skin lesions	Reduces risk of cardiovascular events, prevents atherosclerosis
Chloroquine	Similar to HCQ, it modulates the immune response	Improves cutaneous manifestations in SLE	Prevents arrhythmias (e.g., atrioventricular block, atrial tachycardia/fibrillation)
Corticosteroids (Systemic)	Potent anti-inflammatory suppresses immune response	Controls acute flares of CLE	Long-term risks include hypertension, dyslipidemia, and increased cardiovascular risk.
Corticosteroids (Topical)	Localized anti-inflammatory action	Reduces localized skin lesions	Minimal systemic side effects
Belimumab	Inhibits B-cell activation and autoantibody production	FDA-approved for SCLE reduces systemic lupus activity	Limited direct cardiac effect, potential indirect benefit by controlling inflammation
Sun Protection	Prevents UV-induced skin lesions	Reduces the severity and occurrence of cutaneous manifestations	Indirect benefit by reducing systemic lupus activity, preventing skin-triggered flare-ups

Antimalarials, particularly hydroxychloroquine (HCQ), are the primary treatment for CLE [46]. HCQ improves skin lesions and has favorable effects on cardiac outcomes in SLE [47]. It has been demonstrated that HCQ reduces the risk of cardiovascular events in SLE by its anti-inflammatory and antithrombotic properties, helping prevent atherosclerosis [48]. Yang et al. studied a cohort of 795 SLE patients receiving treatment with HCQ [49]. The results reflected a significant reduction in the risk of coronary heart disease in patients treated with HCQ for at least 318 days. Likewise, a low HR of coronary heart disease was found in patients who received a cumulative dose of HCQ of at least 100.267 mg [49]. Rúa-Figeroa et al. conducted an extensive multicenter retrospective study in a cohort of 117 SLE patients; the findings revealed that administering antimalarials was associated with a decreased incidence of coronary heart disease [50]. An observational study performed by Teixeira et al. found a significant association between the use of chloroquine in patients diagnosed with SLE and the lack of atrioventricular block [51]. Holter monitoring in these patients exhibited an absence of atrial tachycardia/fibrillation in patients with a higher frequency of chloroquine.

Corticosteroids, often used for acute flares of CLE, provide rapid control of inflammation but carry long-term risks, including hypertension, dyslipidemia, and an increased risk of cardiovascular disease [52-54]. However, other studies did not find a significant association [55-57]. Farina et al. described the factors associated with cardiovascular events in a cohort of individuals with SLE, revealing that patients with cardiovascular events were exposed to higher doses of glucocorticoids [58].

Role of Immunosuppressive Therapy and Biologics

In cases of severe or refractory CLE, immunosuppressive therapies such as methotrexate, azathioprine, and mycophenolate mofetil are commonly used. These agents target systemic inflammation and are beneficial for both cutaneous and cardiac manifestations by controlling immune overactivity [59]. Mycophenolate mofetil is effective in reducing skin flares and the risk of lupus myocarditis, demonstrating the dual benefit of these drugs in managing both cutaneous and cardiac disease [60].

Biologic therapies are an emerging class of treatments that have revolutionized the management of refractory SLE, including CLE and associated systemic manifestations. Belimumab, a monoclonal antibody that inhibits B-cell activating factor (BAFF), has been approved for SLE and has shown efficacy in reducing skin lesions. Recent studies have highlighted its role in preventing cardiac involvement [61]. A study has reported that belimumab significantly reduced cardiac flares, including pericarditis and myocarditis, in patients with active SLE, underscoring the importance of biologics in preventing systemic complications linked to skin involvement [62].

Another promising biologic is anifrolumab, which targets type I interferon, a key driver of SLE pathogenesis. Anifrolumab has shown positive results in improving both cutaneous and systemic disease activity in lupus, including reducing cardiovascular inflammation [63]. Ongoing trials are exploring its long-term effects on reducing cardiac morbidity in SLE patients. Casey et al. observed improvements in cholesterol efflux capacity and reductions in GlycA levels among patients with moderate to severe SLE who participated in the MUSE trial and were treated with anifrolumab [64]. The authors suggest that these changes in subclinical cardiovascular disease markers could theoretically indicate a reduced cardiovascular risk in these patients. However, they also acknowledge a significant limitation of the study: these markers are not direct clinical indicators of cardiovascular disease. In a separate longitudinal cohort trial, cardiovascular risks were assessed in SLE patients who had recently initiated treatment with mycophenolate mofetil, cyclophosphamide, or azathioprine [65]. In a cohort of 1,360 patients, a comparison of mycophenolate versus cyclophosphamide therapy showed no statistically significant difference during the first cardiovascular event [65]. In another group of 3,742 patients, the effect of mycophenolate versus azathioprine was evaluated, and the only statistically significant finding was a lower risk of cardiovascular events in the mycophenolate group compared to the azathioprine group in the intention-to-treat analysis over a 12-month period [65].

Multidisciplinary Management of SLE with Skin and Cardiac Involvement

Given the interplay between cutaneous and cardiac manifestations in SLE, a multidisciplinary approach to management is crucial. Dermatologists, rheumatologists, and cardiologists must collaborate to monitor and treat skin and heart involvement patients. Early recognition of cutaneous markers, such as SCLE or vasculitis, by dermatologists can prompt closer cardiovascular surveillance. Dermatologists play a key role in guiding the overall therapeutic approach, especially when cutaneous activity correlates with systemic flares.

Rheumatologists responsible for overall SLE management must incorporate cardiovascular risk assessment into routine care, especially in patients with active skin disease. Regular cardiovascular monitoring (6-12 months) through echocardiograms and electrocardiograms is essential for early detection of subclinical heart involvement. Cardiac screening is particularly important in patients with severe cutaneous lesions, as studies have shown that such patients have a higher incidence of silent cardiovascular complications [66]. Wind et al. conducted a retrospective study to evaluate the effectiveness of a multidisciplinary clinical pathway for managing patients with SLE and/or antiphospholipid syndrome [66]. The study included 78 patients with a total of 112 pregnancies. The multidisciplinary team comprised obstetrics, nephrology, rheumatology, thrombosis, and hemostasis specialists, with additional consultations from cardiologists and other professionals as needed. Although the results indicated a fivefold reduction in the risk of SLE flares, this finding was not statistically significant [66].

Cardiologists, in turn, contribute by managing cardiovascular risk factors, such as hypertension, dyslipidemia, and atherosclerosis, which are exacerbated in SLE patients. A multidisciplinary approach ensures that treatment decisions related to immunosuppressive therapy, biologics, or cardiovascular management are integrated and tailored to the individual patient's risk profile.

Challenges and gaps in current research

Despite significant advancements in understanding the role of cell death pathways in the pathogenesis of SLE, several challenges and gaps remain. One of the primary hurdles is the complexity of the crosstalk between various cell death mechanisms, such as apoptosis, necroptosis, pyroptosis, and autophagy, and their distinct roles in different organ systems. While studies have explored individual pathways, the interactions between these processes in cutaneous and cardiac manifestations of SLE are not yet fully elucidated. This gap limits the development of targeted therapeutic strategies that can modulate these pathways effectively.

Another significant challenge is the heterogeneity of SLE itself. The disease manifests differently across individuals, with variations in severity, organ involvement, and response to therapy. This heterogeneity complicates the task of linking specific cell death pathways to distinct clinical manifestations, such as skin and cardiac involvement. Additionally, most studies rely on animal models or in vitro systems, which, while valuable, do not fully capture the complexity of human SLE.

Further, the role of environmental factors, epigenetic modifications, and genetic predispositions in modulating these cell death pathways remains underexplored. Identifying specific biomarkers that could predict or correlate with the activation of particular cell death pathways in SLE patients would be crucial for advancing personalized medicine in this field.

Lastly, clinical trials focusing on interventions that target cell death pathways are limited. There is a pressing need for more robust clinical studies to validate experimental findings and assess the efficacy of potential therapeutic agents to modulate these pathways in patients with cutaneous and cardiac SLE manifestations.

## Conclusions

Clinically, cutaneous manifestations, particularly subacute and chronic forms of lupus erythematosus, may serve as markers of increased cardiovascular risk, highlighting the need for integrated clinical management. Early recognition of skin lesions could prompt closer cardiovascular monitoring, potentially preventing severe complications. Future research should aim to clarify the precise mechanisms linking skin and heart involvement in SLE, explore novel therapeutic approaches targeting these pathways, and emphasize multidisciplinary care to improve outcomes for patients suffering from these dual manifestations.
